# Patterns of genetic structuring in the coral *Pocillopora damicornis *on reefs in East Africa

**DOI:** 10.1186/1472-6785-9-19

**Published:** 2009-08-26

**Authors:** Petra Souter, Oskar Henriksson, Niklas Olsson, Mats Grahn

**Affiliations:** 1 Australian Institute of Marine Science, PMB No 3, Townsville MC, Queensland 4810 Australia; 2 School of Life Sciences, Sodertorn University College, 141 89 Huddinge, Sweden

## Abstract

**Background:**

Studies of population genetic structures provide an indication of direction and magnitude of larval transport and hence are an important component in the assessment of the ability of reefs to recover from severe disturbance. This paper reports data on population genetic structures in the coral *Pocillopora damicornis *from 26 reefs in Kenya and Tanzania.

**Results:**

Gene flow among reefs was found to be variable, with a significant overall genetic subdivision (*F*_*ST *_= 0.023 ± 0.004 SE; p < 0.001), however, only 34% of all pairwise population comparisons showed significant differentiation. Panmixia could not be rejected between reefs separated by as much as 697 km, while other sites, separated by only a single kilometre, were found to be significantly differentiated. An analysis of molecular variance indicated that population genetic differentiation was significant only at the smaller spatial scale (< 10 km), whereas panmixia could not be rejected between groups of samples separated by over 100 km. Estimates of contemporary gene flow showed similar results, with numbers of first generation migrants within each population ranging from 0 to 4 (~5% of the total number of colonies sampled) and likely dispersal distances ranging between 5 and 500 km.

**Conclusion:**

This study showed that population differentiation in *P. damicornis *varied over spatial scales and that this variability occurred at both evolutionary and ecological time scales. This paradox is discussed in light of stochastic recruitment and small scale population structures found in other species of coral. The study also identifies potential source reefs, such as those within Mnemba Conservation area near Zanzibar and genetically isolated reefs such as those within Malindi Marine National Park and Reserve in northern Kenya.

## Background

Current threats to coral reefs, such as elevated sea water temperatures, coral disease, pollution and destructive and unsustainable fishing methods, have depleted or degraded more than half of the world's coral reefs [1-4]. The poor condition of many of these reefs is attributable to the extreme El Ninõ event in 1998, which functionally destroyed approximately 16% of the world's coral reefs through bleaching-induced mass mortality. In 2004, slightly more than 40% of the reefs affected by the El Ninõ showed signs of recovery. Most of these reefs were exposed to minimal anthropogenic influence either because they were situated within well-managed marine reserves or were geographically remote [2,5]. A prerequisite for such recovery however, is successful recruitment of new coral colonies from remnant source populations. A range of statistics that are based on individual genotypes rather than population level allele frequencies are now available [6,7], which allow the assignment or exclusion of genotypes within sampled populations and hence provide a more ecologically relevant measure of connectivity [8,9]. Hence the results from population genetic studies provides information that can be incorporated into marine protected areas management strategies [10].

To date, the processes underlying the highly variable population genetic structures in corals remain poorly understood [11-14]. Many studies have shown that life history characteristics such as reproductive mode and larval competency may serve as reliable predictors of dispersal patterns in marine invertebrates including corals [9,15-17]. However, other studies have revealed that neither reproductive mode [16,18] nor larval competency [19] are reliable predictors of levels of genetic connectivity. To further complicate matters, many coral species display a range of sexual and asexual reproductive modes that may vary geographically even within the same species [20]. Also, complex morphological species boundaries [21,22], hybridisation [23,24] and cryptic speciation (Souter in review) all contribute to make population genetic studies in corals a daunting task.

*Pocillopora damicornis *is a common scleractinian coral, found on tropical coral reefs throughout the Indo-Pacific region [25]. It has been extensively studied and its reproductive modes and population genetic structures are well documented in many geographic locations. As with its pocilloporid congener *Seriatorpora hystrix *[21] its morphological species status is currently under question, with two genetically distinct but morphologically indistinguishable types (F and NF) occurring in sympatry on reefs in the Western Indian Ocean (Souter 2009 in review). It is a hermaphroditic species, which can reproduce both sexually and asexually [26-28]. Sexual reproduction occurs primarily by internal brooding and release of mature planulae, although broadcast spawning has been reported from Western Australia [28] and the Eastern Pacific [29]. Its asexual reproduction is thought to occur primarily through ameiotic production of brooded larvae, parthenogenesis [26]. Population genetic studies of *P. damicornis *indicate that populations that are influenced by asexual reproduction are less panmictic [30-32] than populations that rely mostly on sexual reproduction [33]. However, small scale sub-divisions have been reported within populations of putative sexual origin in the Eastern Indian Ocean [34] and at Lord Howe Island [35]. In general, dispersal of asexual planulae has been calculated to occur at spatial scales of up to 1 km [30], whereas gene flow between sexually reproducing populations has been reported over distances of up to 1200 km [33]. Interestingly, sexually reproducing populations have been found to show more differentiation at small spatial scales, such as between reef habitats, than over distances of thousands of km [33,35,36]. However, long distance dispersal between reefs on the GBR and high latitude coral reefs further south is apparently exceedingly rare [35]

Like most pocilloporid corals, *P. damicornis *exhibits low tolerance to elevated sea surface temperatures [37,38] and on surveyed reefs along the coast of Kenya more than 75% (at some reefs 100%) of all colonies suffered bleaching-induced mortality as a consequence of the extreme El Ninõ event in 1998 [39,40]. However, pocilloporid corals are effective colonisers of available space on reefs and recent studies indicate that pocilloporid larvae dominate settlement on artificial settlement tiles in the Mombasa Marine National Park and Reserve [41,42] and new recruits (colonies between 0.5 and 5.0 cm in diameter) were found along the entire coast of Kenya by 2004 [5]. As an effective colonist, this species is important to study in relation to recovery of degraded reefs in East Africa.

The coast of East Africa displays a mixture of fringing-, rock island- and patch-reefs. From Malindi to Kisite in Kenya, the coast is lined by an almost unbroken fringing reef, behind which extensive lagoonal reefs are found [43]. Further south, along the coast of Tanzania, the reefs are patchier and rock island reefs are found around the islands of Pemba, Zanzibar and Mafia.

This study was implemented with the aim of examining population genetic patterns in the NF-type of *P. damicornis *at different spatial scales along the coast of East Africa. Levels of connectivity were inferred at evolutionary and contemporary time scales and related to geographic distance among and between reefs as well as the physical location and habitat of the sampled populations. Due to the lack of basic ecological data relating to the reefs included in this study, the underlying project was of an exploratory nature and not hypothesis driven. As a result the discussion is organised around results rather than addressing specific issues or hypothesis.

## Results

**Figure 1:**
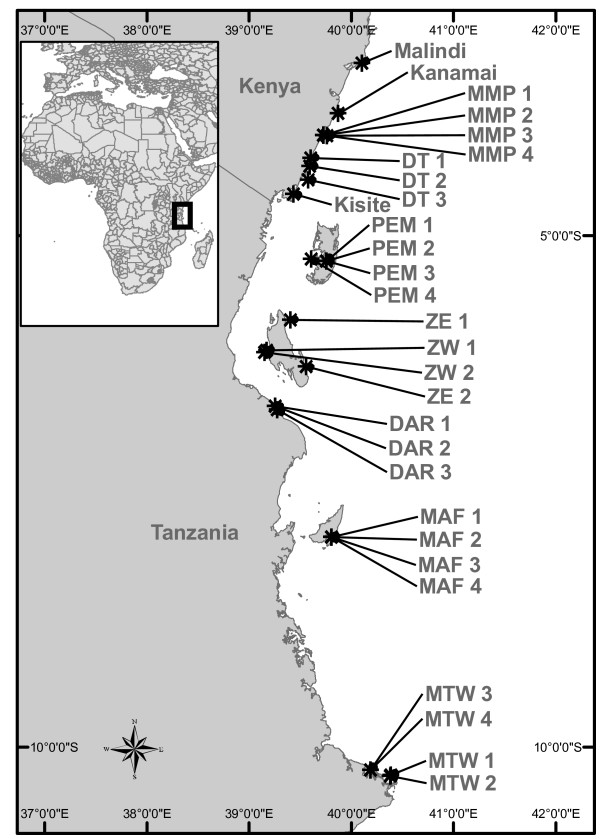
**Map of sample sites**. See Table 1 for groupings and names of sites.

From an initial 29 sites and 825 genotyped colonies, 661 NF-type colonies from 26 sites were included in the full study (Figure 1; Table 1). Two loci (PV 6 and Pd3_002) showed evidence of containing null alleles at 3 and 9 sites respectively. However, as use of the corrected dataset did not significantly alter the resulting F-statistics (Table 2), the original data set was retained to enable comparisons with the results from the exclusion tests. Significant overall genotypic linkage disequilibrium was found between locus PV6 and Pd2_006 and between PV7 and Pd3_005. At the population level, this disequilibrium was only significant in 2 (PV6 – Pd2_006) or 1 (PV7 – Pd3_005) of the 26 populations. *F*_*ST *_outlier tests revealed a signature of positive selection in locus Pd3_002 (probability that simulated *F*_*ST *_< sample *F*_*ST *_= 0.999). However, all significance tests for the F-statistics were done by bootstrapping over loci and the exclusion of locus Pd3_002 from the analysis did not alter the results. As a result, Pd3_002 was included in the study to maximise the power of the analyses. The ratio of unique multi locus genotypes to sampled colonies equalled 1.0 within all sampled populations but an identical multi-locus genotype was shared between DT 2 and MTW3 (see table 1 for reference to site locations). Numbers of alleles per loci ranged between 8 and 24 (average number = 13.17; ± 5.74 sd), and all loci displayed a significant population differentiation (*F*_*ST*_) and a significant deficit of heterozygotes (except Pd3_005) (Table 2). Site specific diversity measures showed similar patterns of high allelic and genetic diversity across all sampled populations. A significant heterozygote deficiency was found in 22 of the 26 sampled populations (Table 1).

**Table 1:** Population statistics (± sd).

**Population**	**N**	**N_NF_**	**A**	**H_E_**	**H_O_**	**F_IS_**
Malindi	35	11	4.83 (± 1.60)	0.66 (± 0.08)	0.45 (± 0.06)	**0.293**

**Mombasa Marine National Park and Reserve (MMP)**						
Bamburi MMP 1	30	30	7.00 (± 2.28)	0.70 (± 0.06)	0.50 (± 0.04)	**0.214**
Coral Gardens MMP 2	34	34	7.83 (± 1.47)	0.69 (± 0.05)	0.52 (± 0.04)	**0.173**
Starfish Gardens MMP 3	35	33	7.00 (± 2.76)	0.69 (± 0.05)	0.55 (± 0.04)	**0.138**
Nyali MMP 4	33	28	6.83 (± 1.83)	0.69 (± 0.07)	0.49 (± 0.04)	**0.195**

Tiwi DT 1	19	19	5.50 (± 2.59)	0.65 (± 0.07)	0.49 (± 0.05)	**0.210**
Diani North end DT 2	30	30	7.67 (± 1.86)	0.72 (± 0.05)	0.48 (± 0.04)	**0.340**
Kisite	29	29	9.17 (± 2.93)	0.73 (± 0.07)	0.60 (± 0.04)	**0.176**

**Pemba Island Chake Bay (PEM)**						
Ataturks PEM 1	29	29	7.00 (± 2.61)	0.72 (± 0.05)	0.55 (± 0.04)	**0.201**
Le Cache PEM 2	23	23	6.50 (± 2.59)	0.68 (± 0.06)	0.47 (± 0.05)	**0.244**
Oh Canada PEM 3	22	22	6.67 (± 1.51)	0.71 (± 0.04)	0.54 (± 0.05)	**0.230**
Anchor chain PEM 4	31	31	7.17 (± 2.04)	0.71 (± 0.05)	0.54 (± 0.04)	0.127

Mnemba ZE 1	27	27	8.67 (± 2.88)	0.78 (± 0.04)	0.52 (± 0.04)	**0.309**
Paje ZE 2	28	27	6.67 (± 2.25)	0.68 (± 0.05)	0.46 (± 0.04)	**0.316**
Kisiwani ZW 1	28	28	7.00 (± 1.90)	0.72 (± 0.04)	0.43 (± 0.04)	**0.297**
Bawi ZW 2	30	30	7.33 (± 2.73)	0.71 (± 0.06)	0.55 (± 0.04)	**0.172**
Mdudya DAR 1	30	22	7.17 (± 2.14)	0.71 (± 0.04)	0.52 (± 0.05)	**0.197**
Bongoyo DAR 3	26	15	6.50 (± 2.17)	0.74 (± 0.07)	0.60 (± 0.05)	**0.214**

**Mafia Island (MAF)**						
Pinnacle MAF 1	29	10	5.00 (± 1.26)	0.70 (± 0.07)	0.62 (± 0.07)	0.052
Maueni Isl. MAF 2	29	25	6.33 (± 1.03)	0.72 (± 0.03)	0.50 (± 0.04)	**0.253**
Jena Reef MAF 3	19	19	6.67 (± 1.63)	0.68 (± 0.05)	0.57 (± 0.05)	0.103
Milimani MAF 4	26	25	7.00 (± 2.19)	0.69 (± 0.04)	0.48 (± 0.04)	**0.255**

**Mtwara**						
Mnazi Bay 1 MTW 1	28	28	6.17 (± 1.83)	0.69 (± 0.03)	0.49 (± 0.04)	**0.298**
Mnazi Bay 2 MTW 2	28	27	7.17 (± 1.60)	0.71 (± 0.04)	0.56 (± 0.04)	**0.147**
Lulu shoals MTW 3	25	20	6.33 (± 2.16)	0.71 (± 0.07)	0.57 (± 0.05)	0.123
Monoliths MTW 4	30	30	7.17 (± 1.72)	0.66 (± 0.06)	0.52 (± 0.04)	**0.192**
Total	825	661	13.60 (± 6.80)	0.74 (± 0.04)	0.55 (± 0.01)	**0.256**

Total number of sampled colonies (N), number of NF-types (N_NF_), average number of alleles per locus (A), expected (H_E_) and observed (H_O_) heterozygotisy. Bold F_IS_-values indicate a significant departure from Hardy-Weinberg equilibrium (p < 0.01). The four groups of populations included in the AMOVA are MMP 1–4, PEM 1–4, MAF 1–4 and MTW 1–4.

**Table 2:** Locus specific statistics (± s.d.).

*Locus*	*No alleles*	*F*_ST_	*F*_IS_	*F_IS_: data corrected for null alleles*
PV6	12	**0.015(± 0.004)**	**0.226 (± 0.022)**	**0.216 (± 0.012)**
PV7	12	**0.032 (± 0.012)**	**0.500 (± 0.038)**	**0.500 (± 0.038)**
Pd3_004	8	**0.022 (± 0.012)**	**0.278 (± 0.031)**	**0.278 (± 0.031)**
Pd3_002	9	**0.036 (± 0.023)**	**0.489 (± 0.035)**	**0.401 (± 0.021)**
Pd3_005	24	**0.024 (± 0.009)**	0.038 (± 0.036)	0.038 (± 0.036)
Pd2_006	14	**0.014 (± 0.006)**	**0.140 (± 0.034)**	**0.140 (± 0.034)**

**Total**	13.17 (± 5.74)	**0.023 (± 0.004)**	**0.258 (± 0.070)**	**0.242 (± 0.056)**

Values in bold show significant deviations from the Hardy-Weinberg equilibrium (p < 0.001)

**Figure 2:**
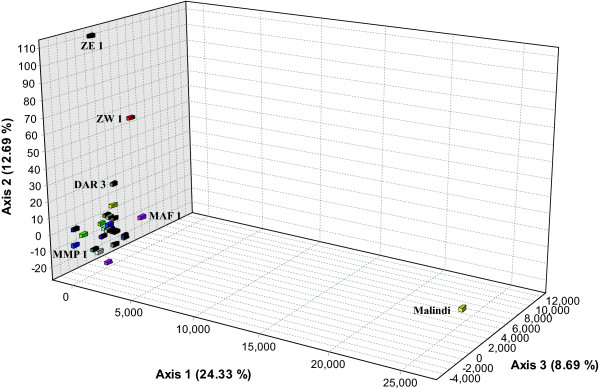
**PCA plot displaying the genetic distance between the samples depicted as the centre of gravity [of] all sampled colonies within a sampled site**.

**Figure 3:**
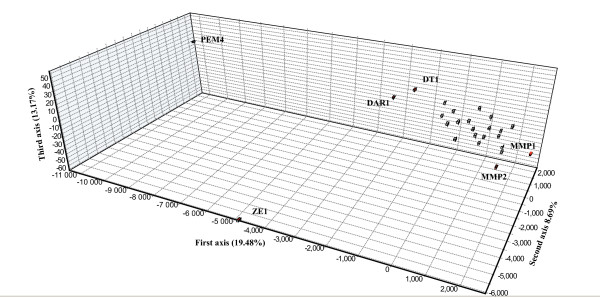
**A PCA plot depicting genetic distances between less divergent samples, constructed by removing the sample from Malindi**.

Between population differentiation accounted for 2.3% of the overall deviation from HWE (total *F*_*ST *_= 0.023 ± 0.004; p < 0.001) (Table 2) with 34% (p < 0.003; α = 0.01) of all pairwise population comparisons showing significant differentiation (Additional file 1). The degree of isolation varied, with some sites (Malindi, MMP 1, ZE 1 and ZW 1) being significantly differentiated from over 88% of all other sites sampled, while others (DT 1 and PEM 2 – 4) were in HWE with 21 of the other 25 sites (Additional file 1). The Principal Component Analysis (PCA) further illustrated the differentiation of Malindi along the first axis, ZE 1 and ZW 1 along the second axis and MMP1 along the third axis, with the addition of MAF 1 (Figure 2). By removing Malindi from this analysis, PEM 4 and ZE 1 were differentiated along the first and second axis (Figure 3). The results of the AMOVA showed that the level of isolation did not increase with increased spatial scales but rather that sample subdivision was greater among sites within groups (*F*_*SG *_0.009; p = 0.002) than among groups (*F*_*GT *_0.001; p = 0.343), with no significant deviation from HWE found at the larger spatial scale (Table 3). Mantel tests of the reduced major axis regression analysis showed no significant correlation between log (genetic) and log (geographic) distance among all sites (R^2 ^= 0.012; p = 0.053), nor among sites within any of the seven groups (p values ranged from 0.33 – 0.83). This remained the case also after excluding highly divergent sites such as Malindi, MMP 1, ZE 1 and ZW 2 (R^2 ^= 0.0015; p = 0.248).

**Table 3:** Results from the AMOVA showing the partitioning of genetic variation among and within groups and individuals.

***Source of variation***	***df***	***Variance component***	***Fixation index***	***p-value***
Among groups (F_GT_)	3	0.001	0.001	0.343
Among sites within groups (F_SG_)	12	0.015	0.009	0.002
Among individuals within sites (F_IS_)	398	0.330	0.190	< 0.001
Within individuals (F_IT_)	414	1.403	0.198	< 0.001

Exclusion tests revealed that 32 colonies (5%) had a probability of less than 0.01 of being derived from within the site at which they were sampled (hence, these were identified as first generation immigrants). Out of these, 14 colonies had a probability of being derived from a genetically divergent, sampled site of > 0.05. Six populations (MMP 1 and 3, DT 1, PEM 2, MAF 1 and MTW 3) showed no signs of recent immigration and Mnemba Conservation Area near Zanzibar (ZE 1) was found to be the most likely source population for six colonies sampled across the entire geographic range of the study (Additional file 2).

Occurrence of F-types (i.e., the colonies belonging to the putative cryptic species that were excluded from the analyses above) showed no consistent pattern or correlation to any obvious difference in habitat and the proportion of the F-types sampled at each site varied from 0 (15 sites) to 100% (Kanamai) (Table 1).

## Discussion

### Population genetic patterns

It is important to emphasise that although a very small amount of migration is enough to counteract genetic drift, not rejecting panmixia does not equate to open populations that are capable of seeding degraded neighbouring or distant reefs. In fact, replenishing severely depleted populations requires hundreds or thousands of successful migrants per generation [44]. Conversely, given enough samples and loci, almost any pair of data will be significantly different unless there is complete panmixia. The impact of cryptic speciation on this study resulted in a somewhat diminished dataset. Naturally this had an affect on the power of the statistics employed, as does the fact that only six microsatellite loci are available and produced acceptable results. Nonetheless, according to simulations by Kalinowski [45], the significance of F_ST _values derived from 6 loci with an average of 13 alleles per loci (>78 independent alleles) results in a coefficient of variation of < 0.25, suggesting that the power of the statistics employed herein with is sufficient.

Due to the difference in polymorphism between allozymes and microsatellites, a direct comparison between reported *F*_*ST *_values from previous spatial genetic studies is inappropriate [46]. However, the variable levels of dispersal that were found between populations examined in this study are consistent with earlier findings from a range of coral species (c.f. [16,47]) and previous studies of genetic connectivity in marine invertebrates from the coast of East Africa, which have all reported gene flow over distances of hundreds of kilometres [48-51]. Small scale population differentiation, such as that found between sites within groups in this study, has previously been reported for this species [32,34,52] and other brooding corals such as *Seriatopora hystrix *[47,53,54] and could be attributed to localised recruitment, due to short dispersal distances of brooded larvae, or site specific selection, which may not be equally apparent when groups of populations are compared over larger distances.

For the purpose of managing coral reef ecosystems, processes at ecological time scales need to be separated from those at evolutionary time scales [13]. This can be achieved by exclusion and assignment tests, which have been found to provide a relevant measure of contemporary gene flow [14,55,56]. As only a minuscule proportion of the total number of populations was sampled for this study, the assignment of individuals to "home" should be interpreted with some caution. With that in mind, results from exclusion tests suggest that contemporary dispersal between populations occurs at both spatial scales. However, it does not occur among or between all sampled populations, which concurs with the variable levels of divergence indicated by the *F*-statistics. Exclusion tests further increased the number of potentially isolated reefs by showing that six reefs show no signs of first generation migrants. MMP 1 was the only site that showed consistent genetic isolation at both evolutionary and contemporary time scales. MMP 1, which is a reef slope site off Bamburi Beach, was isolated from nearby lagoonal populations, corroborating previous results for the massive coral *Platygyra daedalea *[51]. Furthermore, and in accordance with what was found in *P. daedalea *[51], MMP 1 was not significantly differentiated from the only other reef slope site sampled along the fringing reef of the Kenyan coast (DT 1). The results from these two studies may indicate that dispersal is limited between the reef slope and lagoon in this region, while gene flow occurs along the outer reef slope. A similar pattern of differentiation between lagoonal and reef slope sites was found by Benzie et al [36] who proposed that differential selection acting at the two habitats could be a plausible explanation. However, as reef slope sites sampled in this study are not consistently differentiated from lagoonal sites and as five out of the six studied loci show no evidence of being functional and hence are not prone to selection, this hypothesis is unlikely to explain the differentiation. Another possible explanation may be found in local hydrodynamic patterns, such as the impact of boundary currents at the reef edge. Boundary currents arise by a decoupling of inshore and offshore currents and are generated on the Great Barrier Reef when wind and current directions are opposed [57]. Such a decoupling would result in limited across shelf mixing, which would hamper larval dispersal across reef shelves, while maintaining dispersal in a north – south direction. However, as no detailed, small scale hydrodynamic data is published from this region, this hypothesis remains un-tested.

A lack of successful dispersal from southerly reefs may explain the divergence of Malindi from other sampled populations. Malindi was found to be genetically isolated from the southerly lagoonal reefs, (with the exception of Kanamai), also in *P. daedalea *[51]. Studies from the Western Indian Ocean suggest that coral reproduction primarily occurs during the northeast monsoon between October and March [58-61]. Along the coast of Kenya this monsoon counteracts and slows down the north flowing East African coastal current, which may hamper long distance dispersal of larvae and give rise to more isolated populations further north. Conversely, results from the exclusion tests indicate that 2 out of 11 colonies may be first generation migrants. With such a high proportion of recent migrants, the inferred level of differentiation between Malindi and other sites may potentially be eroded, assuming that the current populations persist. A more extensive data set, which includes additional reefs in this area, would undoubtedly reveal more information regarding this genetic break and may shed light on the underlying ecological or physiological processes that drive this divergence.

Mnemba conservation area on the northeast tip of Unguja Island, Zanzibar (ZE 1) is genetically differentiated from all sites except DAR 3 and MAF 1, yet is the most likely source population for 6 of the 31 detected first generation migrants in the data set. This population has the highest genetic diversity, and hence has an increased likelihood of being perceived as the source population of colonies harbouring alleles that are rare or absent in other populations. Another population on Zanzibar (ZW 1) is equally isolated, indicating that these two sites, along with MMP 1, harbour predominantly self seeding populations.

Surveys conducted after the 1998 coral bleaching event showed that reefs off the coast of Dar es Salaam were not significantly affected by the bleaching; in fact, hard coral cover increased between 1997 and 1999 [62]. Despite this obvious difference in recent bottleneck and founder events, these populations (DAR 1 and 3) show a similar level of genetic diversity and variable levels of differentiation to the recently depleted populations in Kenya, with no significant differentiation between the two sites and genetic similarity to a majority of the other sampled sites.

All four sites from Mafia are sampled within Chole Bay and within a kilometre of each other. No pairwise comparisons within the group showed significant differentiation, nor are they significantly differentiated to other sampled sites with the exception of the six isolated sites discussed earlier. No comparative data from before and after the 1998 coral bleaching event is available for Chole Bay on Mafia. However, hard coral cover was reported to be 30% in 1999, which would suggest that bleaching had not significantly affected these reefs [62].

Despite the relatively large geographic distance between the sites at Mtwara and the other reefs, significant differentiation was only found between these sites and the isolated sites of Malindi, MMP 1, ZE 1 and ZW 1 as well as DT 2. Hence, sufficient gene flow to counteract the effects of random genetic drift seemingly occurs at spatial scales of up to 697 km (between Mtwara and Mombasa marine national park and reserve). The exclusion test further reveals that first generation migrants are likely to have dispersed from Mtwara to Zanzibar, a distance of over 500 km. If dispersal occurs primarily through a stepping stone model, a significant isolation by distance should, in theory, be apparent. However, this was not found to be the case for this dataset. Significant isolation by distance has been detected at spatial scales similar to those between sites within groups (1 – 50 km) for the brooding corals *Balanophyllia elegans *[63] and *Seriatopora hystrix *[53]. The results obtained from the present study revealed a more disordered genetic structure with no significant correlations between genetic and geographic distances at either of the two spatial scales. Such "chaotic genetic patchiness" is commonly reported in marine invertebrates. This phenomena is explained by factors such as pre- or post-settlement selection and different genetic origins of settling larvae [64]. Indeed, long distance dispersal, coupled with site specific selection or small scale hydrodynamic patterns, would serve to explain the fact that distant groups of samples, separated by over 100 km, show a higher degree of similarity than sites within groups that are sampled at geographic scales of 0.5 – 10 km. At larger spatial scales, a lack of correlation between genetic and geographic distance may be attributed to a higher impact of genetic drift, whereas at very small spatial scales gene flow may not be sufficiently unidirectional to cause a significant correlation [13]. Further support for the chaotic genetic patchiness theory is derived from the heterozygote deficiencies and population specific linkage disequilibria. A majority of the sampled populations and loci showed a significant deficit of heterozygotes. The exception to this finding is locus Pd3_005 (the most polymorphic locus), which appears to be in HWE. As the presence and potential impact of null alleles could not explain this large deficit across all loci and populations, other theoretical explanations are needed. In most coral genetic studies, a heterozygote deficit is explained by inbreeding and non-random mating [49,65]. However, a heterozygote deficiently coupled with high genetic diversity and population specific linkage disequilibrium may indicate recent admixture. Indeed, several recent studies have incorporated admixture and spatial and temporal variability of larval sources in the explanation [50,55,66] and it is widely accepted that population genetic structure can be affected by temporal variability in recruitment sources and rates [55,67-69]. The variable impact of coral bleaching on these reefs (see for example [40]) may well cause a temporal shift in source populations as population sizes fluctuate over time. Hence, the results of this study, which includes multiple cohorts and populations impacted by recent founder events, is likely to be affected by spatially and temporally variable recruitment events, which in turn would cause a deficit of heterozygotes across both loci and populations. Also, many locations and reefs remain un-sampled and the structure of the hierarchal sampling design would be greatly improved by increasing the number of sites at the smaller geographical scales. Despite this, the data provides important information from many previously un-studied reef areas and is the most comprehensive study of population genetic patterns of reef building corals in the Western Indian Ocean to date.

### The F-types

In a separate study, colonies of *P. damicornis *were genetically characterised at one nuclear and two mitochondrial sequence markers and six microsatellite loci. Both genomes support the existence of two reciprocally monophyletic clusters of WIO origin indicative of two reproductively isolated species within *P. damicornis*. Some interesting observations regarding the 154 colonies that were found to belong to the F-types and hence excluded from the population genetic statistics are worth reporting here. The most striking difference related to the prevalence of asexual reproduction, which would suggest that the two types differ in their preferred, or most commonly utilised, reproductive mode. Among the F-types, 105 colonies out of 154 were found to belong to 14 clonal lineages (Table 4), while among the NF-types, only a single identical multi-locus genotype was encountered among the 661 samples.

**Table 4:** Identical multi-locus genotypes of the F-type and their associated probability of being sexually produced (p_sex_) based on global allele frequencies and the total number of colonies associated (n).

***Clone no***	***PV 7***		***PV 6***		***Pd3***	***_004***	***Pd3***	***_002***	***Pd3***	***_005***	***Pd2***	***_006***	***p*_*sex*_**	***n***	***Site***
1	224	224	192	206	159	162	184	199	213	213	197	197	1.41E-11	10	Malindi/Kanamai
2	224	224	192	204	159	162	184	199	216	216	197	197	0.0003	4	Malindi/Kanamai
3	224	224	192	206	162	168	184	184	213	213	195	195	0.001	4	Kanamai
4	224	224	192	206	159	162	184	184	213	213	195	195	0.0006	4	Kanamai
5	224	224	206	206	159	162	184	184	207	208	195	199	2.75E-26	21	DT 3
6	224	224	206	206	159	162	184	196	207	213	195	199	0.001	5	DT 3
7	224	224	204	204	159	162	196	199	210	224	195	195	4.61E-17	6	DAR 3
8	224	224	198	202	159	159	184	199	213	233	195	195	2.09E-35	7	DAR1
9	224	224	204	204	159	162	184	184	213	224	195	195	4.66E-28	20	DAR 2
10	224	224	200	204	159	159	187	187	213	222	195	195	5.50E-11	5	MAF 1
11	224	224	204	204	159	159	187	199	213	213	195	195	3.42E-23	13	MAF 1/MAF 4
12	224	224	204	204	159	162	184	184	207	213	195	199	3.12E-05	2	MAF 2
13	224	224	202	202	159	159	199	199	213	213	195	195	2.61E-05	2	MAF 2
14	224	224	202	202	162	162	184	184	230	233	195	195	1.58E-06	2	MTW 3

Also, the fact that no NF-types were sampled at Kanamai reef indicates a lack of successful recruitment onto this reef. The lagoon at Kanamai is very shallow and has the most extreme temperature variations recorded among Kenyan lagoonal reefs [70]. During low tide, much of the reef is exposed or submerged in water that reaches temperatures of above 34°C. Unlike other surveyed reefs, Kanamai lagoon showed no significant decline in hard coral cover following the extreme El Ninõ event of 1998 [70]. In addition, it hosted the only recorded, remnant population of *P. damicornis *along the coast of Kenya [71], which may suggest that the F-types are better adapted to the prevailing conditions at Kanamai.

## Conclusion

In summary, the spatial genetic patterns reported in this study indicate a variable degree of isolation of populations at both ecological and evolutionary time scales, with certain sites showing a high degree of connectivity and others relying mostly on self seeding. The results highlight the importance of identifying and protecting reefs that harbour high levels of genetic biodiversity and that act as potential source reefs, such as Mnemba conservation area on Zanzibar. It also provided further evidence that certain reef slope sites are unique, such as MMP 1, which was shown to be isolated from closely positioned lagoonal populations in the Mombasa marine national park and reserve in both the present study as well as previous studies of another species of coral (*Platygyra daedalea*). Without a doubt, the incorporation of ecological, hydrodynamic and temporal data into the spatial genetic studies would provide a more comprehensive picture of reef connectivity in this region and improve management decisions and conservation efforts. Unfortunately, such data is yet largely unavailable for most of the reefs in the WIO region despite the vital role these ecosystems play both as biodiversity hot spots as well as by providing essential goods and services for rapidly growing coastal towns and communities.

## Methods

### Sites

Samples of *P. damicornis *were collected from a total of 29 sites along the coasts of Kenya and Tanzania, from Malindi marine national park and reserve (3°18'35S; 40°06'57E) to Mtwara marine national park (10°11'33 S; 40°12'04 E), spanning a distance of approximately 860 km (Figure 1). A majority of the collections in Kenya were made in shallow (< 5 m) back reef lagoons except sites at Bamburi and Tiwi, which were collected from the reef slope at a depth of 10 – 15 m. Samples from Pemba, ZE 1 and 2, MTW 3 and 4 and MAF 3 were collected at a depth of 10 – 15 m. All other samples from Tanzania were collected on shallow reefs (< 5 m). The total area sampled varied depending on the abundance of the species at the site. Most commonly the collections were made along a transect ranging between 100 – 500 m in length. In order to investigate various scales of connectivity, a nested sampling design was used in four areas (Mombasa Marine National Park and Reserve, Pemba Island, Mafia Marine National Park and Mtwara). The four areas were separated by between 120 – 700 km and the four sites within each area were separated by 0.7 – 10 km.

### Sample collection and preparation

A small fragment (~1 cm) was cut off a central branch of 19 – 35 colonies at each site, making a total of 825 samples. Samples were collected at least five metres from their nearest sampled neighbour to minimise the inclusion of clones that were a result of fragmentation, and were kept at ambient temperature in 70% ethanol until further processing.

DNA was extracted using the Qiagen^® ^DNEasy kit according to a modified protocol for rodent tails (fragments were placed directly into lysis buffer and Proteniase K and kept in a water bath at 56°C over night). A polymerase chain reaction (PCR) was carried out using six fluorescently labelled microsatellite primers developed for *Pocillopora *spp.: PV 6 and PV 7 (Magalon et al. 2004b), and Pd3_002, Pd3_004, Pd2_006, and Pd3_005 (Starger et al. 2008). The PCR was conducted in 10 μl reactions using 25 ng of DNA, 0.25 U AmpliTaq^© ^(Applied Biosystems), and a concentration of 0.25 mM of each dNTP, 0.1 mM of MgCl_2 _and 0.4 mM of each primer. The thermal cycling protocol was initiated with 5 minutes at 95°C followed by 30 × (30 s at 95°C; 30 s at 53°C (PV 6 & PV 7) or 58°C (Pd3_002, Pd3_004, Pd2_006 and Pd3_005) and 1 min at 72°C) and ended with a 10 minute extension at 72°C. Non amplifying samples were re-run at a 5°C lower annealing temperature than those stated above. PCR products as well as positive and negative controls were visualised on an ABI Prism 3700 DNA Analyzer (ABI, Applera Cooperation) together with a GeneScan 500-Rox ladder and genotyped automatically and verified manually using GeneMapper^© ^version 4.0-software (ABI, Applera Cooperation).

### Data analysis

Due to the prevalence of two genetically distinct but morphologically similar types (F and NF) being present on reefs in East Africa (Souter, in review), the population genetic statistics presented in this study are based only on the NF-type, which was found in sufficient numbers on 26 out of the 29 sampled reefs. All downstream calculations are based on 661 NF-type individuals from 26 populations. The sample from Kanamai was entirely made up of F-types and only 3 and 6 individuals from DT 3 and DAR 2 respectively were of the NF-type, hence those three sites were excluded from further analysis.

Resulting genotypes were checked for scoring errors and potential null alleles using Micro-Checker [72]. Loci were also tested for linkage disequilibrium (LD) using Arlequin v 3.1.1 [73] and signatures of selection by comparing locus-specific *F*_*ST*_-values to 10,000 simulated *F*_*ST*_-values according to the FST-outlier method using the Selection Workbench [74].

To avoid over-estimating genetic divergence between populations, the microsatellite Excel toolkit [75] was used to identify identical multi-locus genotypes that were likely to be a result of asexual reproduction within each population. The toolkit was also used to infer levels of genetic diversity, measured as observed (*H*_*O*_) and expected levels of heterozygosity (*H*_*E*_), according to Nei [76] and average numbers of alleles per locus and population.

The programme Fstat [77] was used to calculate allele frequencies (Appendix 1), and inbreeding coefficients partitioned among individuals within sample (*F*_*IS*_), sites within total (*F*_*ST*_), and individuals within total (F_*IT*_), according to Weir and Cockerham [78]. By using this test statistic, the differences in sample sizes are considered as allele frequencies are weighted according to sample size. Significant genetic differentiation between population pairs was detected after correction using the false discovery rate (FDR) [79] as presented by Narum [80] (α = 0.01). To test the spatial scales of connectivity, an analysis of molecular variance (AMOVA) within (< 10 km) (*F*_*SG*_) and between (> 100 km) (*F*_*GT*_) four groups of samples was done using the Arlequin software (see Figure 1 and Table 2 for details on groups). The software Genetix [81] was used to construct a principal component analysis (PCA) to visualise the genetic distance between genotypes using each locus as an independent binary variable according to She et al[82]. As a large number of individuals were genotyped, the output of the PCA was simplified by plotting the centre of gravity of all colonies within a sample (cendroid), as proposed by the authors of the software. A second PCA was constructed after removing the site at Malindi to visualise the partition among the remaining cluster of samples.

According to a review by Jones et al [14], exclusion test implemented by the programme GeneClass v 2.0 [6] has shown promising results regarding inferring contemporary gene flow. The test statistic used for this data set was the likelihood of the individual genotype originating within the population where the individual was collected (L-home), which is the appropriate statistic to use when not all possible source populations have been sampled [6]. Assignment tests using a partial Bayesian have been verified to be an accurate prediction of dispersal, especially when levels of natal dispersal are less than 18% [56]. Hence, first generation migrants were inferred using the partial Bayesian criterion according to Rannala and Mountain [83] which was compared with the distribution of likelihoods for 10,000 simulated genotypes created by a Monte Carlo algorithm according to Paetkau et al. [7]. Individuals with a genotype that showed a probability of < 0.01 of being generated within their sampled population were considered immigrants. A possible source population was identified as the sample with the highest likelihood of being "home" to the genotype. Re-assignments were only made if the likelihood of home had a higher probability than 0.05 and if the putative source population was significantly differentiated from the sampled population.

The isolation by distance web service (IBDWS) [84] was used to estimate the correlation between the genetic and logarithmic geographic distance by a reduced major axis (RMA) regression. Significance values were obtained through a Mantel test using 1000 randomisations. This test was conducted twice, once including all samples and once excluding the most divergent sites (Malindi, MMP 1, ZE 1 and ZW 2).

## Authors' contributions

The project was conceptualised by PS, who acquired the funding in collaboration with MG. Collections and laboratory work were done by PS, OH and NO. OH implemented an initial pilot study for this project as his undergraduate thesis work under the supervision of PS and MG. Interpretation of the data and writing of the manuscript was done by PS. MG supervised PS during her PhD work and revised earlier versions of the manuscript.

## Supplementary Material

Additional file 1 Table of genetic and geographic distances. Click here for file

Additional file 2 Table of migrants. Click here for file
