# Supplementary material for: Patterns of genetic structuring in the coral Pocillopora damicornis on reefs in East Africa
Source: BMC Ecol. 2009 Aug 26;9:19. doi: 10.1186/1472-6785-9-19 (PMC2751742; doi:10.1186/1472-6785-9-19)
Supplement: Additional file 1 — Table of genetic and geographic distances. [file 1472-6785-9-19-S1.doc]

| **Additional file 1**. Table of genetic (lower left) and geographic (upper right) distances. Genetic distance is calculated as pairwise F_ST_ and geographic distance is given in km.  Bold figures indicate significant genetic differentiation (p < 0.003; α = 0.01 Boxes represent groups of samples. | | | | | | | | | | | | | | | | | | | | | | | | | | |
| --- | --- | --- | --- | --- | --- | --- | --- | --- | --- | --- | --- | --- | --- | --- | --- | --- | --- | --- | --- | --- | --- | --- | --- | --- | --- | --- |
|  | **Malindi** | **MMP 1** | **MMP 2** | **MMP 3** | **MMP 4** | **DT 1** | **DT 2** | **Kisite** | **PEM1** | **PEM 2** | **PEM 3** | **PEM 4** | **ZE 1** | **ZE 2** | **ZW1** | **ZW2** | **DAR1** | **DAR 3** | **MAF 1** | **MAF 2** | **MAF 3** | **MAF 4** | **MTW 1** | **MTW 2** | **MTW 3** | **MTW 4** |
| **Malindi** |  | 87.2 | 87.9 | 88.3 | 93 | 110.6 | 124.4 | 174.1 | 214.4 | 217.8 | 220.3 | 223.8 | 290.8 | 336.3 | 328.9 | 333.1 | 383.7 | 387.7 | 514.7 | 513.6 | 514.1 | 513.5 | 771.1 | 771.2 | 771.3 | 771.4 |
| **MMP 1** | **0.174** |  | 1 | 1.6 | 5.9 | 23.4 | 37.2 | 86.9 | 130.6 | 133.9 | 136.6 | 140.2 | 206.1 | 254.4 | 243.2 | 247.4 | 299.6 | 303.7 | 436.5 | 435.4 | 436 | 435.4 | 697.1 | 697.2 | 697.3 | 697.4 |
| **MMP 2** | **0.171** | **0.020** |  | 0.7 | 5.2 | 22.7 | 36.5 | 86.3 | 130.2 | 133.5 | 136.3 | 139.8 | 205.7 | 254.1 | 242.7 | 246.9 | 299.1 | 303.3 | 436.3 | 435.1 | 435.8 | 435.1 | 696.9 | 696.9 | 696.9 | 696.9 |
| **MMP 3** | **0.180** | **0.032** | 0.006 |  | 4.7 | 22.3 | 36.1 | 85.9 | 129.9 | 133.3 | 136 | 139.5 | 205.4 | 253.8 | 242.4 | 246.5 | 298.9 | 303 | 436.1 | 435 | 435.6 | 435 | 696.8 | 696.8 | 696.8 | 696.8 |
| **MMP 4** | **0.166** | **0.026** | -0.003 | 0.005 |  | 17.6 | 31.4 | 81.2 | 125.6 | 128.9 | 131.7 | 135.2 | 200.9 | 249.5 | 237.8 | 242 | 294.4 | 298.6 | 431.9 | 430.8 | 431.5 | 430.8 | 692.9 | 692.1 | 692.11 | 692.12 |
| **DT 1** | **0.190** | 0.019 | -0.002 | 0.010 | 0.004 |  | 13.8 | 63.7 | 109.6 | 113 | 115.8 | 119.3 | 184.3 | 233.7 | 220.8 | 225 | 277.8 | 288.1 | 416.9 | 415.8 | 416.4 | 415.8 | 678.6 | 678.7 | 678.8 | 678.9 |
| **DT 2** | **0.148** | **0.034** | 0.024 | **0.021** | 0.025 | 0.028 |  | 49.8 | 96.9 | 100.3 | 103.1 | 106.6 | 171.1 | 221 | 207.2 | 211.4 | 264.5 | 268.8 | 404.6 | 403.5 | 404.2 | 403.6 | 666.9 | 666.1 | 666.11 | 666.12 |
| **Kisite** | **0.164** | **0.029** | 0.012 | 0.011 | 0.024 | 0.024 | 0.001 |  | 55.6 | 58.6 | 61.5 | 64.7 | 124 | 176.2 | 158.6 | 162.7 | 217 | 221.4 | 361.1 | 360 | 360.7 | 360.1 | 624.9 | 624.1 | 624.11 | 624.12 |
| **PEM 1** | **0.174** | **0.013** | 0.002 | **0.014** | 0.011 | 0.007 | **0.022** | 0.008 |  | 3.4 | 6.2 | 9.7 | 76.4 | 124.1 | 115.3 | 119.6 | 169.4 | 173.4 | 307.9 | 306.7 | 307.4 | 306.8 | 570.7 | 570.8 | 570.9 | 570.1 |
| **PEM 2** | **0.198** | **0.049** | 0.010 | 0.009 | 0.004 | 0.017 | 0.011 | 0.026 | 0.018 |  | 2.9 | 6.4 | 73.1 | 120.8 | 112.1 | 116.4 | 166 | 170.1 | 304.6 | 303.4 | 304.1 | 303.5 | 567.5 | 567.6 | 567.7 | 567.8 |
| **PEM 3** | **0.144** | **0.026** | 0.009 | 0.007 | 0.013 | 0.013 | 0.001 | 0.003 | -0.005 | 0.004 |  | 3.5 | 70.7 | 117.9 | 109.9 | 114.2 | 163.5 | 167.5 | 301.7 | 300.6 | 301.2 | 300.6 | 564.6 | 564.7 | 564.8 | 564.9 |
| **PEM 4** | **0.183** | **0.032** | 0.001 | 0.003 | 0.012 | 0.011 | 0.011 | 0.003 | -0.005 | 0.003 | -0.006 |  | 67.3 | 114.4 | 106.6 | 110.9 | 160 | 164 | 298.2 | 297.1 | 297.7 | 297.1 | 561.2 | 561.3 | 561.4 | 561.5 |
| **ZE 1** | **0.158** | **0.067** | **0.064** | **0.074** | **0.079** | **0.076** | **0.029** | **0.037** | **0.047** | **0.067** | **0.035** | **0.051** |  | 55 | 40.1 | 44.4 | 93.5 | 97.8 | 239.1 | 237.9 | 238.7 | 238.1 | 503.9 | 503.1 | 503.11 | 503.12 |
| **ZE 2** | **0.177** | **0.038** | -0.005 | 0.012 | 0.008 | 0.000 | **0.027** | 0.025 | 0.003 | -0.005 | 0.000 | -0.005 | **0.064** |  | **0.031** | 47.6 | 53.2 | 55.6 | 185 | 183.9 | 184.7 | 184 | 449.5 | 449.6 | 449.7 | 449.8 |
| **ZW 1** | **0.168** | **0.050** | **0.034** | **0.030** | **0.033** | **0.035** | **0.051** | **0.038** | **0.017** | **0.045** | **0.026** | **0.021** | 0.045 | 47.1 |  | 4.3 | 60.6 | 65.4 | 213.9 | 212.8 | 213.7 | 213.1 | 478.4 | 478.5 | 478.6 | 478.7 |
| **ZW 2** | **0.174** | **0.039** | 0.006 | 0.008 | 0.014 | 0.016 | **0.014** | 0.016 | 0.007 | 0.000 | 0.003 | 0.002 | **0.047** | 0.001 | **0.032** |  | 57.2 | 62.1 | 211.1 | 210 | 210.1 | 210.3 | 475.4 | 475.5 | 475.6 | 475.7 |
| **DAR 1** | **0.170** | **0.026** | 0.004 | 0.011 | 0.015 | 0.004 | **0.009** | 0.007 | -0.008 | 0.000 | -0.015 | -0.009 | 0.040 | -0.005 | **0.029** | -0.001 |  | 5 | 154.9 | 153.7 | 154.7 | 154 | 418.5 | 418.6 | 418.7 | 418.8 |
| **DAR 3** | **0.154** | **0.031** | 0.016 | 0.023 | 0.020 | **0.039** | 0.011 | 0.027 | 0.009 | 0.020 | 0.004 | 0.010 | 0.025 | 0.011 | **0.027** | 0.009 | 0.018 |  | 149.9 | 148.8 | 149.7 | 149.1 | 413.5 | 413.6 | 413.7 | 413.8 |
| **MAF 1** | **0.142** | **0.040** | 0.017 | 0.009 | 0.020 | 0.020 | 0.005 | 0.014 | 0.009 | 0.001 | -0.015 | -0.002 | 0.034 | 0.005 | 0.028 | 0.027 | -0.006 | 0.017 |  | 1.1 | 0.9 | 1.2 | 265 | 266 | 267 | 268 |
| **MAF 2** | **0.150** | **0.026** | 0.001 | 0.013 | 0.006 | 0.001 | **0.032** | **0.033** | 0.005 | 0.005 | 0.001 | 0.006 | **0.062** | -0.012 | **0.026** | 0.006 | 0.002 | 0.013 | 0.007 |  | 1.2 | 0.8 | 266.1 | 266.2 | 266.3 | 266.4 |
| **MAF 3** | **0.187** | **0.033** | 0.003 | 0.018 | 0.006 | 0.014 | **0.042** | 0.039 | 0.006 | 0.003 | 0.008 | 0.008 | **0.075** | -0.014 | **0.035** | 0.002 | 0.007 | 0.010 | 0.019 | -0.007 |  | 0.7 | 265.3 | 265.3 | 265.3 | 265.3 |
| **MAF 4** | **0.169** | **0.016** | 0.001 | 0.005 | 0.001 | 0.013 | 0.006 | 0.003 | -0.002 | 0.001 | -0.010 | -0.005 | **0.050** | 0.006 | **0.027** | 0.010 | -0.006 | 0.010 | -0.006 | 0.013 | 0.006 |  | 265.9 | 265.9 | 265.9 | 265.9 |
| **MTW 1** | **0.189** | **0.028** | 0.003 | 0.004 | 0.002 | 0.016 | **0.010** | 0.006 | -0.001 | -0.001 | -0.003 | -0.010 | **0.048** | 0.003 | **0.025** | 0.002 | -0.002 | 0.002 | -0.002 | 0.008 | 0.006 | -0.011 |  | 5 | 5 | 2 |
| **MTW 2** | **0.165** | **0.039** | 0.001 | 0.020 | 0.005 | 0.013 | **0.044** | 0.035 | 0.009 | 0.018 | 0.011 | 0.010 | **0.065** | -0.007 | **0.034** | 0.009 | 0.013 | 0.020 | 0.017 | -0.007 | -0.003 | 0.023 | 0.012 |  | 2 | 5 |
| **MTW 3** | **0.154** | **0.017** | 0.003 | 0.017 | 0.002 | 0.011 | **0.027** | 0.022 | 0.003 | 0.011 | 0.008 | 0.010 | **0.061** | 0.003 | **0.028** | 0.007 | 0.003 | 0.024 | 0.019 | 0.004 | -0.006 | 0.001 | 0.009 | 0.014 |  | 5 |
| **MTW 4** | **0.204** | **0.047** | 0.014 | 0.002 | 0.013 | 0.023 | **0.012** | 0.007 | 0.011 | 0.002 | 0.000 | -0.002 | **0.069** | 0.012 | **0.042** | 0.005 | -0.006 | 0.032 | 0.010 | 0.024 | 0.021 | -0.003 | -0.001 | 0.028 | 0.016 |  |
