# Supplementary material for: Patterns of genetic structuring in the coral Pocillopora damicornis on reefs in East Africa
Source: BMC Ecol. 2009 Aug 26;9:19. doi: 10.1186/1472-6785-9-19 (PMC2751742; doi:10.1186/1472-6785-9-19)
Supplement: Additional file 2 — Table of migrants. [file 1472-6785-9-19-S2.doc]

| **Additional file 2.** Table of migrants. Number of 1^st^ generation migrants in each population (N_M_) inferred as the likelihood of the genotype originating at sampled location being < 0.01. Numbers are the probability of sample originating in a particular population. If no sampled and significantly differentiated population gave a probability of > 0.05 of being the source population, the migrant remained un-assigned. Numbers along diagonal are the proportion of individuals sampled at each site that originated from that site (self recruitment). | | | | | | | | | | | | | | | | | | | | | | | | | | | |
| --- | --- | --- | --- | --- | --- | --- | --- | --- | --- | --- | --- | --- | --- | --- | --- | --- | --- | --- | --- | --- | --- | --- | --- | --- | --- | --- | --- |
|  | **N_M_** | **M**  **A**  **L** | **M**  **M**  **P**  **1** | **M**  **M**  **P**  **2** | **M**  **M**  **P**  **3** | **M**  **M**  **P**  **4** | **DT 1** | **DT 2** | **K**  **I**  **S** | **PEM1** | **PEM2** | **PEM3** | **PEM4** | **ZE 1** | **ZW1** | **ZE 2** | **ZW2** | **DAR1** | **DAR3** | **M**  **A**  **F**  **1** | **M**  **A**  **F**  **2** | **M**  **A**  **F**  **3** | **M**  **A**  **F**  **4** | **M**  **T**  **W**  **1** | **M**  **T**  **W**  **2** | **M**  **T**  **W**  **3** | **M**  **T**  **W**  **4** |
| **MAL** | 2 | *0.81* |  |  |  | 0.76 |  |  | 0.70 |  |  |  |  |  |  |  |  |  |  |  |  |  |  |  |  |  |  |
| **MMP 1** | 0 |  | *1.0* |  |  |  |  |  |  |  |  |  |  |  |  |  |  |  |  |  |  |  |  |  |  |  |  |
| **MMP 2** | 2 |  |  | *0.94* |  |  |  |  |  |  |  |  |  |  |  |  |  |  |  |  |  |  |  |  |  |  |  |
| **MMP 3** | 0 |  |  |  | *1.0* |  |  |  |  |  |  |  |  |  |  |  |  |  |  |  |  |  |  |  |  |  |  |
| **MMP 4** | 2 | 0.19 |  |  |  | *0.93* |  |  |  |  |  |  |  |  |  |  |  |  |  |  |  |  |  |  |  |  |  |
| **DT 1** | 0 |  |  |  |  |  | *1.0* |  |  |  |  |  |  |  |  |  |  |  |  |  |  |  |  |  |  |  |  |
| **DT 2** | 4 |  |  |  |  |  |  | *0.87* |  |  |  |  |  | 0.67 |  |  |  |  |  |  |  |  |  |  |  |  |  |
| **Kisite** | 4 |  |  |  |  |  |  |  | *0.86* |  |  |  |  |  |  |  |  |  |  |  | 0.29 |  |  |  |  |  |  |
| **PEM 1** | 1 |  |  |  |  |  |  |  |  | 0.96 |  |  |  | 0.40 |  |  |  |  |  |  |  |  |  |  |  |  |  |
| **PEM 2** | 0 |  |  |  |  |  |  |  |  |  | *1.0* |  |  |  |  |  |  |  |  |  |  |  |  |  |  |  |  |
| **PEM 3** | 1 |  |  |  |  |  |  |  |  |  |  | *0.95* |  | 0.05 |  |  |  |  |  |  |  |  |  |  |  |  |  |
| **PEM 4** | 1 |  |  |  |  |  |  |  |  |  |  |  | *0.97* |  |  |  |  |  |  |  |  |  |  |  |  |  |  |
| **ZE 1** | 1 |  |  |  |  |  |  |  |  |  |  |  |  | *0.96* | 0.67 |  |  |  |  |  |  |  |  |  |  |  |  |
| **ZW 1** | 2 |  |  |  |  |  |  |  |  |  | 0.65 |  |  |  | *0.93* |  |  | 0.20 |  |  |  |  |  |  |  |  |  |
| **ZE 2** | 1 |  |  |  |  |  |  |  |  |  |  |  |  | 0.62 |  | *0.96* |  |  |  |  |  |  |  |  |  |  |  |
| **ZW 2** | 1 |  |  |  |  |  |  |  |  |  |  |  |  | 0.48 |  |  | *0.97* |  |  |  |  |  |  |  |  |  |  |
| **DAR 1** | 1 |  |  |  |  |  |  |  |  |  |  |  |  |  |  |  |  | *0.95* |  |  |  |  |  |  |  |  |  |
| **DAR 3** | 1 |  |  |  |  |  |  |  |  |  |  |  |  |  |  |  |  |  | *0.93* |  |  |  |  |  |  |  |  |
| **MAF 1** | 0 |  |  |  |  |  |  |  |  |  |  |  |  |  |  |  |  |  |  | *1.0* |  |  |  |  |  |  |  |
| **MAF 2** | 1 |  |  |  |  |  |  |  |  |  |  |  |  |  |  |  |  |  |  |  | *0.96* |  |  |  |  |  |  |
| **MAF 3** | 1 |  |  |  |  |  |  |  |  |  |  |  |  |  |  |  |  |  |  |  |  | *0.95* |  |  |  |  |  |
| **MAF 4** | 2 |  |  |  |  |  |  |  |  |  |  |  |  |  |  |  |  |  |  |  |  |  | *0.92* |  |  |  |  |
| **MTW 1** | 2 |  |  |  |  |  |  |  |  |  |  |  |  |  |  |  |  |  |  |  |  |  |  | *0.93* |  |  |  |
| **MTW 2** | 1 |  |  |  |  |  |  |  |  |  |  |  |  | 0.65 |  |  |  |  |  |  |  |  |  |  | *0.96* |  |  |
| **MTW 3** | 0 |  |  |  |  |  |  |  |  |  |  |  |  |  |  |  |  |  |  |  |  |  |  |  |  | *1.0* |  |
| **MTW 4** | 1 |  |  |  |  |  |  |  |  |  |  |  |  |  |  |  |  |  |  |  |  |  |  |  |  |  | *0.97* |
| **TOT** | **32** | **1** | **0** | **0** | **0** | **1** | **0** | **0** | **1** | **1** | **1** | **0** | **0** | **6** | **1** | **0** | **0** | **1** | **0** | **0** | **1** | **0** | **0** | **0** | **0** | **0** | **0** |
